# Evaluation of the LDBio ICT IgG/IgM lateral flow assay versus the Bordier Elisa assay for the diagnosis of chronic pulmonary aspergillosis in Nigeria

**DOI:** 10.1128/spectrum.01533-24

**Published:** 2025-02-06

**Authors:** O. J. Balogun, R. O. Oladele, O. O. E. Ajibola, A. A. Davies, T. A. Fashanu, A. O. Nwosu, B. E. Ekeng, J. P. Gangneux

**Affiliations:** 1 Department of Biomedical Engineering, University of Lagos, Lagos, Nigeria; 2 Medical Mycology Society of Nigeria, Lagos, Nigeria; 3 Department of Medical Microbiology and Parasitology, Faculty of Basic Medical Sciences, College of Medicine, University of Lagos, Lagos, Nigeria; 4 Department of Systems Engineering, Faculty of Engineering, University of Lagos, Lagos, Nigeria; 5 Department of Medical Microbiology and Parasitology, Olabisi Onabanjo University Teaching Hospital, Sagamu, Nigeria; 6 Department of Medical Microbiology and Parasitology, University of Calabar Teaching Hospital, Calabar, Nigeria; 7 Centre National de Référence des Mycoses et Antifongiques LA-AspC Aspergilloses Chroniques, European Excellence Center for Medical Mycology (ECMM EC), Centre Hospitalier Universitaire de Rennes, Rennes, France; 8 Univ Rennes, CHU Rennes, Inserm, EHESP, Irset (Institut de Recherche en Santé, Environnement et Travail), UMR_S 1085, Rennes, France; Brown University, Providence, Rhode Island, USA; Nicolae Testemitanu State University of Medicine and Pharmacy, Chișinău, Moldova

**Keywords:** chronic pulmonary aspergillosis, point-of-care diagnosis, antibodies, *Aspergillus fumigatus*, *Aspergillus flavus*

## Abstract

The detection of anti-*Aspergillus* antibodies is key for diagnosing chronic pulmonary aspergillosis (CPA). Available techniques are limited and pose a considerable challenge in resource-limited settings. The objective of this study was to evaluate the performance of the point-of-care test LDBio ICT IgG/IgM lateral flow assay (LDBio) versus that of the Bordier Elisa assay in a field study in Nigeria. Of the 97 serum samples tested in the CPA patient group, 71 tested positive by the LDBio assay with 69.4% sensitivity. In the non-CPA group, 286 of the 289 sera tested negative by the LDBio assay with 98.7% specificity. The Bordier test was positive for 97 of the 97 CPA serum samples tested, showing 100% sensitivity, significantly different from the LDBio test in detecting *Aspergillus* antibodies (*P* < 0.0001). However, there was no significant difference in the specificity between the two tests (*P* = 0.617). The results were in agreement for 353 of the 386 samples tested (91.5%), with a Cohen’s kappa coefficient of 0.75, indicating substantial agreement between the LDBio and Bordier test results. The LDBio lateral flow assay (LFA) test is a simple and rapid point-of-care test that can be used in field studies in which the Elisa test is not available. The lower sensitivity of the LDBio LFA in our field study could have been due to *A. fumigatus* being the species involved in only 50%–60% of cases in Nigeria, with *A. flavus* being more frequent than in Northern countries.

**IMPORTANCE:**

Available techniques for the detection of *Aspergillus* IgG are limited and pose a considerable challenge in resource-limited settings in terms of affordability, skilled personnel, equipment, and a regular power supply. A point-of-care test would address most of these challenges. The LDBio lateral flow assay (LFA) test is a simple and rapid point-of-care test that can be used in field studies in which the Elisa test is not available. When combined with clinical features, the LFA can be used as a screening tool for chronic pulmonary aspergillosis in settings such as ours; however, a lower sensitivity was observed compared to the Elisa.

## INTRODUCTION

The gold standard for the diagnosis of chronic pulmonary aspergillosis (CPA) requires the detection of circulating *Aspergillus*-specific antibodies (1). CPA is an indolent progressive disease that affects immunocompetent (2) and mildly immunosuppressed individuals (3). It is a sequela mainly following active or previous tuberculosis, non-tuberculosis mycobacterial infections, chronic obstructive pulmonary disease, sarcoidosis, or prior lung surgery (4). It is caused primarily by *Aspergillus* species and affects pre-existing lung cavities (5), with *A. fumigatus* being the most common cause (2). The highest burden of pulmonary tuberculosis (PTB) occurs in Africa and Asia and it is the highest major risk factor for CPA (5), with an estimated global prevalence of 1.2 million cases (6). Because the clinical signs of CPA are non-specific and the symptoms similar to those of PTB (7), its diagnosis requires the presence of respiratory and/or constitutional symptoms for at least 3 months, radiological abnormalities, and serological or microbiological evidence of *Aspergillus* (8). Although serology is important/critical for the detection of CPA, most commercial serological assays primarily detect antibodies against *A. fumigatus*. However, CPA can be caused by other *Aspergillus* species and, thus, false-negative results can occur (7).

Available serological assays show varying performance (9). One method, a semi-quantitative assay (8), identifies precipitin antibodies (7) with a specificity of 100% (10) and a sensitivity of 89.3% (11), but the test lacks standardization and shows poor inter-laboratory reproducibility (12). It has been superseded by a quantitative automated enzyme-linked immunosorbent assay (Elisa) (10). However, the Elisa is expensive and requires skilled personnel, making it not readily available in low and middle-income countries (LMIC). The lateral flow assay, a simple point-of-care test based on immunochromatography, is easy to perform and can be carried out at the bedside, with a turnaround time of 20–30 min (13). We evaluated the performance of the LDBio ICT IgG/IgM lateral flow assay (LFA) versus that of the Bordier Elisa in a field study in Nigeria.

## MATERIALS AND METHODS

We carried out a comparison study using stored serum samples of 97 confirmed cases of CPA (according to the ERS/ESCMID guidelines) from three sites in Nigeria (14). All patients had previously been diagnosed and managed for pulmonary tuberculosis (14, 15).

### Definitions

– CPA was diagnosed using the criteria for resource-constrained countries, which requires the following combinations: (i) >1 symptom that has persisted for ≥3 months, such as hemoptysis, persistent cough, weight loss, and/or breathlessness; (ii) radiological abnormalities showing progressive cavitation, pericavitary infiltrates, and/or pleural thickening, and/or fungal ball; (iii) microbiological evidence of a positive *Aspergillus* IgG antibody testing and/or *Aspergillus* hyphae or *Aspergillus* growth on sputum culture; and (iv) exclusion of other alternative diagnoses (7).
– Pulmonary tuberculosis was diagnosed in patients with positive smear microscopy, culture, or GeneXpert (Cepheid) for *Mycobacterium tuberculosis* from a biological specimen (14, 15).

Serum samples from 289 healthy blood donors from nine sites representing the six geopolitical zones of Nigeria in a study to standardize *Aspergillus* IgG levels in Nigeria (16) were also included in the study. All samples were stored at −80°C prior to running the assay. In total, 386 samples already tested with the Bordier Elisa kit were tested using the *Aspergillus* ICT IgG LFA (LDBio Diagnostics, Lyon, France).

The test kits were stored at 4°C after they were received. For the ICT IgG LFA, a box of 10 cartridges per pack was allowed to warm up to room temperature prior to use, and each test was performed strictly based on the manufacturer’s instructions. Fifteen microliter of serum was added to the sample port of the cartridge, followed by four drops of the elution buffer. The results were read after 30 min and interpreted based on the manufacturer’s insert. The result of the ICT was read visually. The presence of a blue line (control) and black line (sample), irrespective of the intensity, indicates that the sample is positive, whereas the presence of a single blue line indicates that the sample is negative.

The serological testing was carried out on the sera using the Bordier Elisa kit, which is used for the quantitative detection of *Aspergillus* IgG. Antibodies present in the sera bind to *Aspergillus* antigen-precoated wells, and non-specific antibodies are removed by washing. The presence of *Aspergillus*-specific antibodies is detected following the addition of a protein A-alkaline phosphatase conjugate. A second washing step removes the unbound conjugate. Addition of the substrate (pNPP) reveals the bound antibodies, as the wells become yellow in the presence of the alkaline phosphatase. The optical density (color intensity) is proportional to the amount of *Aspergillus fumigatus*-specific antibodies in the sample when read at 405 nm.

### Statistical analysis

Data are reported using frequency-distribution plots (bar plots) for categorical variables. McNemar’s test (DTComPair of the R package) was used with Yates correction (1.0) for the pairwise comparison of the sensitivity of the LDBio and Bordier test kits. Youden’s *J* statistic (sensitivity + specificity − 1) and the diagnostic odds ratio were calculated for the two tests (17). The binomial confidence interval (CI) (95%) was calculated for the sensitivity and specificity of the tests (18). We compared the results of the LDBio and Bordier tests by determining the global concordance for all samples tested and estimated the strength of agreement using Cohen’s kappa coefficient with the following interpretation: poor (0–0.20), fair (0.21–0.40), moderate (0.41–0.60), good (0.61–0.80), and very good (0.81–1). Receiver operating curves (ROCs) of the LDBio and Bordier tests were plotted, and the areas under the curve (AUCs) were evaluated to compare the performance of the LDBio and Bordier tests at varying thresholds. A two-tailed *P* value ≤0.05 was considered statistically significant for all results. R Statistical Computing Software version 4.3.1 was used for data analysis.

## RESULTS

**Fig 1:**
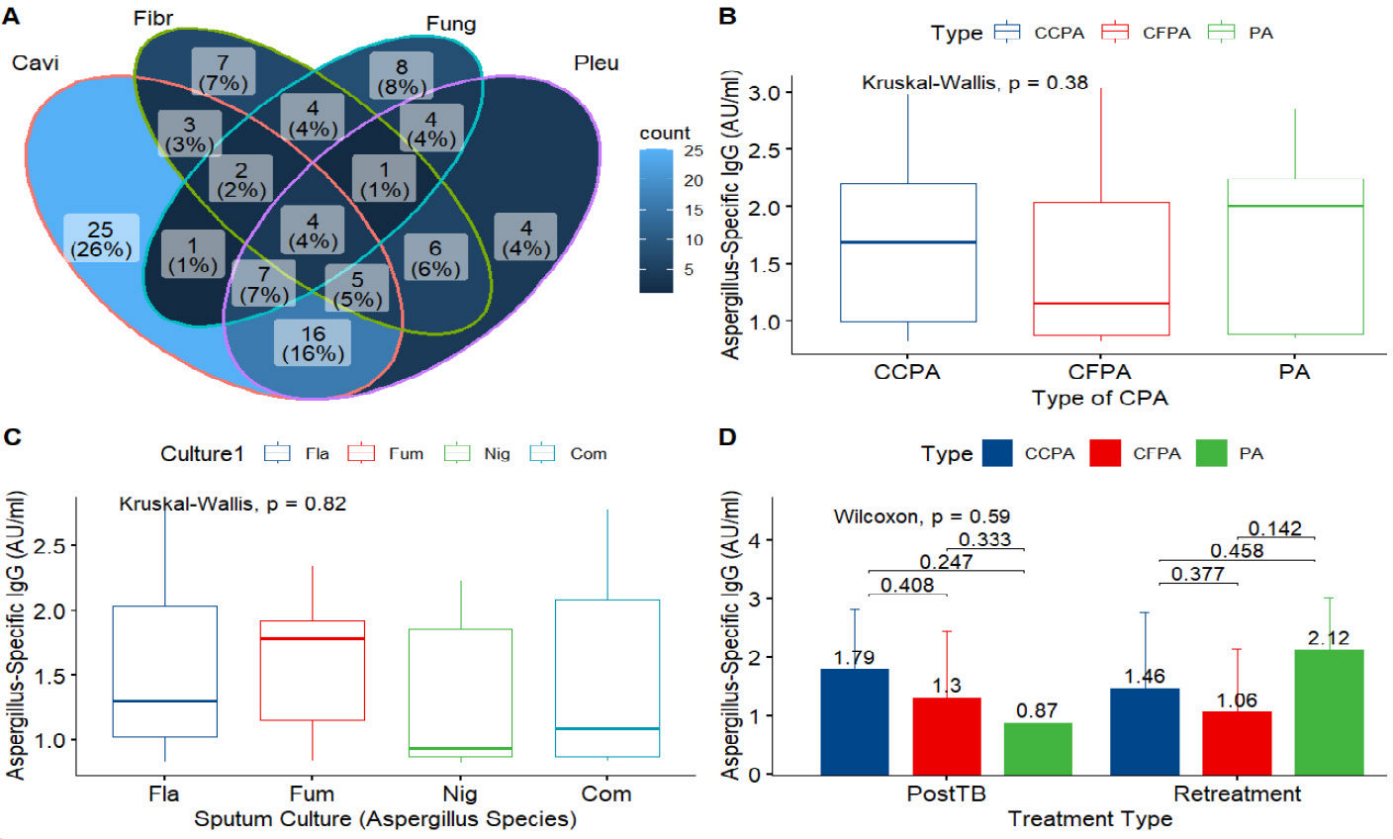
Chest X-ray findings and *Aspergillus* IgG distribution according to CPA type. (**A**) Prevalence of CPA radiological features. Prevalence of only one feature, two, three, and all four of cavitation (Cavi), fibrosis (Fibr), fungal ball (Fung), and pleural thickening (Pleu) were 45.4%, 35.1%, 15.5%, and 4.1% respectively. (**B**) Distribution of *Aspergillus*-specific IgG levels in type of CPA. *Aspergillus* IgG levels during chronic cavitary pulmonary aspergillosis (CCPA), chronic fibrosing pulmonary aspergillosis (CFPA), and pulmonary aspergilloma (PA) were 1.689 (0.992–2.206) AU/mL, 1.154 (0.884–3.034) AU/mL, and 2.01 (0.888–2.856) AU/mL, respectively. (**C**) Distribution of *Aspergillus* IgG levels according to the species: *Aspergillus flavus;* IgG level = 1.303 (1.024–2.036) AU/mL, *Aspergillus fumigatus;* IgG level = 1.781 (1.154–1.922) AU/mL, *Aspergillus niger;* IgG level = 0.933 (0.872–1.855) AU/mL, and *Aspergillus* IgG level for two or three combination of *Aspergillus* species (Com) = 1.089 (0.873–2.082) AU/mL. (**D**) Distribution of *Aspergillus*-specific IgG in the stratification of treatment type and type of CPA. *Aspergillus*-specific IgG level in post-TB group = 1.446 (0.976–2.168) AU/mL, in retreatment group = 1.367 (0.874–2.197) AU/mL.

**Fig 2:**
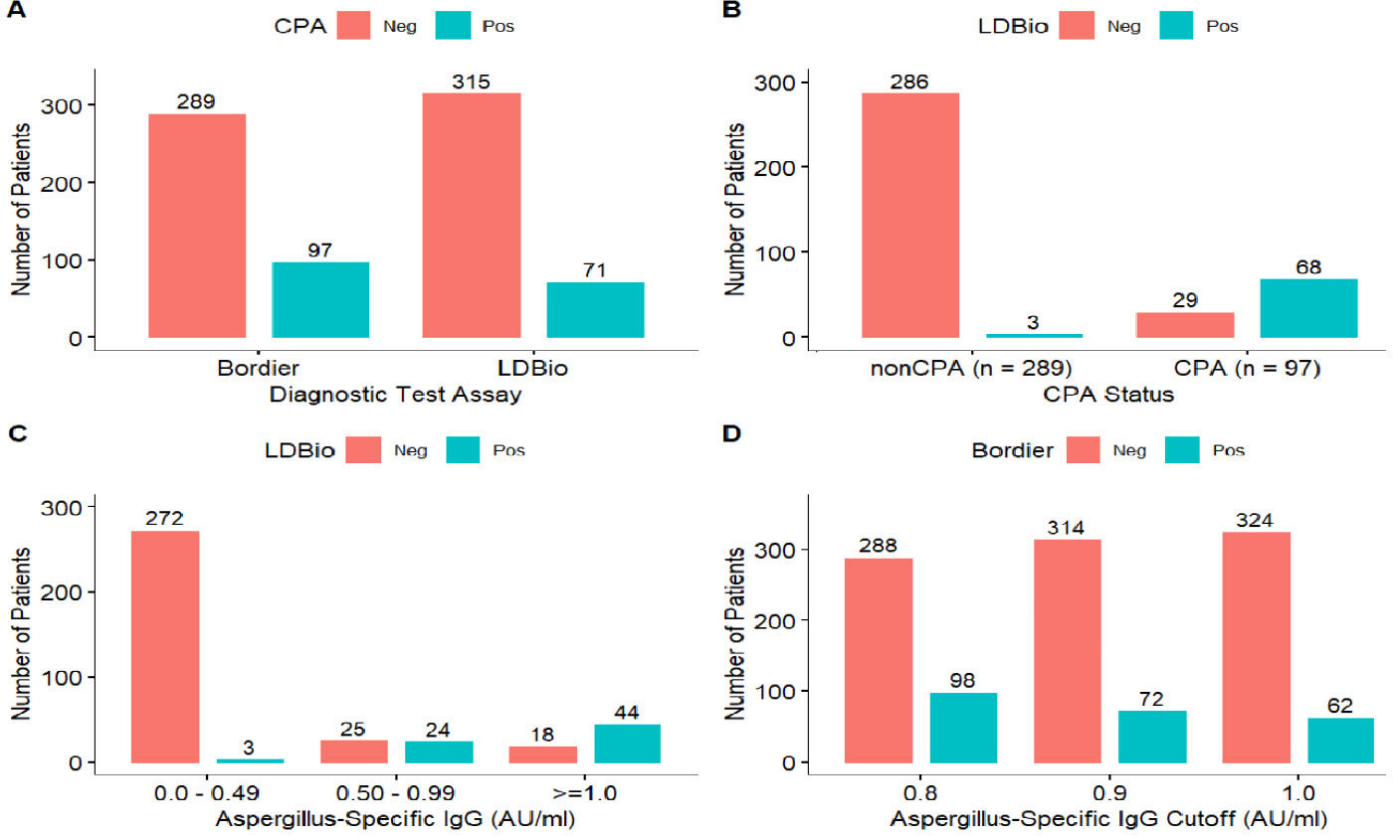
Diagnostic results of Bordier test kits (the gold standard) and the LDBio test (Index test). (**A**) Distribution of the frequency of positive results for the Bordier (97 CPA) and LDBio (71 CPA) tests. (**B**) Distribution of the frequency of misclassification for the LDBio test for CPA (29 misclassified as non-CPA) and non-CPA (three misclassified as CPA) patients. (**C**) Distribution of the frequency of Bordier *Aspergillus*-specific IgG titers for the sera of 386 patients categorized as low (0–0.49 AU/mL), medium (0.5–0.99 AU/mL), or high (≥1.0 AU/mL) tested with the LDBio *Aspergillus* ICT test. (**D**) Distribution of the frequency of Bordier *Aspergillus* IgG titers for cutoffs of 0.8, 0.9, and 1.0 AU/mL for 386 patients tested with the LDBio test.

Of the 386 patient samples tested with the Bordier test kit, 98 patients had positive *Aspergillus* IgG levels (≥0.8 AU/mL). Among them, 97 had radiological features consistent with CPA, i.e., 25.1% (97/386) of the population (Tables 1 and 2; Fig. 1A and 2A). Regarding the tuberculosis (TB) status, 62 (64%) had retreatment TB and 35 (36%) were post-TB patients (Table 1). Positive sputum culture for *Aspergillus* was achieved in 41 (42%) of the patients (Table 2). The majority of *Aspergillus* species was *Aspergillus flavus* (11%), followed by *Aspergillus niger* (10%). The symptoms were not associated with the type of CPA (CCPA, chronic cavitary pulmonary aspergillosis; CFPA, chronic fibrosing pulmonary aspergillosis; PA, pulmonary aspergilloma), except for breathlessness score that has a statistically significant association (*P* = 0.005). The stratification of chest X-ray findings with type of CPA revealed that bronchiectasis and collapse were not statistically significant (all *P* > 0.05) while the remaining chest X-ray features were statistically significantly associated with type of CPA (all *P* < 0.05) (Table 2). The distribution of the characteristic chest X-ray findings of CPA showed prevalence of 44/97 (45.4%) in only one radiological feature, 34/97 (35.1%) prevalence in any two radiological features of fungal ball, fibrosis, cavitation, and pleural thickening, 15/97 (15.5%) in any three, and 4/97 (4%) in any four (Fig. 1A). Although, no statistically significant difference was seen in the pairwise comparison of post-TB and retreatment and in *Aspergillus* IgG level stratified by type of CPA or by *Aspergillus* species (Fig. 1B through D). However, there was a large difference in the aspergilloma IgG level in the post-TB group (0.87 AU/mL) compared to the retreatment group (2.12 AU/mL) (Fig. 1D).

**TABLE 1:** Sociodemographic and clinical characteristics of the CPA patients

	Type of CPA, *N* (%)					
Parameters	*N*	Overall, *N* = 97	CCPA, *N* = 45	CFPA, *N* = 44	PA, *N* = 8	*P* value^*a*^
Sex	97					0.461
Female		43 (44%)	22 (49%)	19 (43%)	2 (25%)	
Male		54 (56%)	23 (51%)	25 (57%)	6 (75%)	
Age (years)	97	40 (29, 52)	38 (26, 50)	41 (31, 52)	48 (41, 51)	0.413
Age group (years)	97					0.946
<21		5 (5.2%)	3 (6.7%)	2 (4.5%)	0 (0%)	
21–30		22 (23%)	12 (27%)	9 (20%)	1 (13%)	
31–40		22 (23%)	10 (22%)	11 (25%)	1 (13%)	
41–50		22 (23%)	9 (20%)	9 (20%)	4 (50%)	
51–60		16 (16%)	7 (16%)	8 (18%)	1 (13%)	
>60		10 (10%)	4 (8.9%)	5 (11%)	1 (13%)	
Employment status	97					0.746
Employed		56 (58%)	28 (62%)	23 (52%)	5 (63%)	
Retired		4 (4.1%)	1 (2.2%)	3 (6.8%)	0 (0%)	
Self-employed		17 (18%)	5 (11%)	10 (23%)	2 (25%)	
Student		10 (10%)	6 (13%)	4 (9.1%)	0 (0%)	
Unemployed		10 (10%)	5 (11%)	4 (9.1%)	1 (13%)	
Treatment type	97					0.41
Post-TB		35 (36%)	17 (38%)	17 (39%)	1 (13%)	
Retreatment		62 (64%)	28 (62%)	27 (61%)	7 (88%)	
Previous TB treatment	97	38 (39%)	14 (31%)	21 (48%)	3 (38%)	0.253
Smoking	97	28 (29%)	15 (33%)	11 (25%)	2 (25%)	0.747
Comorbidity	97	32 (33%)	8 (18%)	20 (45%)	4 (50%)	0.009

^*a*^ Fisher's exact test, Kruskal-Wallis rank sum test.

**TABLE 2:** Sputum culture, symptoms, and radiological findings in CPA patients

	Type of CPA, *N* (%)					
Parameters	*N*	Overall, *N* = 97	CCPA, *N* = 45	CFPA, *N* = 44	PA, *N* = 8	*P* value^*b*^
Productive cough	97	70 (72%)	31 (69%)	33 (75%)	6 (75%)	0.885
Hemoptysis	97	40 (41%)	15 (33%)	23 (52%)	2 (25%)	0.132
Chest pain	97	43 (44%)	23 (51%)	16 (36%)	4 (50%)	0.336
Fever	97	37 (38%)	20 (44%)	14 (32%)	3 (38%)	0.509
Night sweat	97	17 (18%)	9 (20%)	5 (11%)	3 (38%)	0.138
Fatigue	97	44 (45%)	19 (42%)	22 (50%)	3 (38%)	0.71
MRCBS^*a*^	97					0.005
<3		57 (59%)	34 (76%)	20 (45%)	3 (38%)	
≥3		40 (41%)	11 (24%)	24 (55%)	5 (63%)	
GeneXpert	97					0.781
Negative		61 (63%)	27 (60%)	30 (68%)	4 (50%)	
Positive		19 (20%)	9 (20%)	8 (18%)	2 (25%)	
Not done		17 (18%)	9 (20%)	6 (14%)	2 (25%)	
Positive sputum culture	97	41 (42%)	18 (40%)	22 (50%)	1 (13%)	0.142
*Aspergillus* species	97					0.125
*A. flavus*		11 (11%)	2 (4.4%)	9 (20%)	0 (0%)	
*A. fumigatus*		7 (7.2%)	2 (4.4%)	5 (11%)	0 (0%)	
*A. niger*		10 (10%)	4 (8.9%)	5 (11%)	1 (13%)	
*A. flavus* and *A. fumigatus*		8 (8.2%)	7 (16%)	1 (2.3%)	0 (0%)	
*A. niger* and *A. flavus*		1 (1.0%)	0 (0%)	1 (2.3%)	0 (0%)	
*A. niger* and *A. fumigatus*		2 (2.1%)	2 (4.4%)	0 (0%)	0 (0%)	
*A. niger, A. flavus,* and *A.* *fumigatus*		2 (2.1%)	1 (2.2%)	1 (2.3%)	0 (0%)	
None		56 (58%)	27 (60%)	22 (50%)	7 (88%)	
Bronchiectasis	97	5 (5.2%)	1 (2.2%)	4 (9.1%)	0 (0%)	0.37
Cavitation	97	63 (65%)	41 (91%)	22 (50%)	0 (0%)	<0.001
Consolidation	97	41 (42%)	17 (38%)	24 (55%)	0 (0%)	0.007
Collapse	97	27 (28%)	13 (29%)	14 (32%)	0 (0%)	0.191
Fibrosis	97	32 (33%)	0 (0%)	32 (73%)	0 (0%)	<0.001
Aspergilloma	97	31 (32%)	0 (0%)	23 (52%)	8 (100%)	<0.001
Opacity	97	51 (53%)	28 (62%)	22 (50%)	1 (13%)	0.028
Pleural thickening	97	47 (48%)	20 (44%)	27 (61%)	0 (0%)	0.003

^*a*^ MRCBS, medical research council dyspnoea scale.

^*b*^ Fisher's exact test.

The LDBio *Aspergillus* ICT LFA was positive for 71 (15.8%) patients (Fig. 2A). The contingency table of the two binary diagnostic tests showed misclassification of one non-CPA subject as a CPA patient for the Bordier test with a cutoff of 0.8 AU/mL (Table S1). Positivity for CPA for the Bordier test was 98 (25.4%) samples for a threshold of 0.8 AU/mL (sensitivity = 100% [95% CI, 96.3%–100%], specificity = 99.7% [95% CI, 98.1%–99.9%], accuracy = 99.74% [95% CI, 98.6%–99.9%]), while positivity for CPA for LDBio test was 68 (17.6%) for a Bordier test threshold of 0.8 AU/mL (sensitivity = 69.4% [95% CI, 59.3%–78.3%], specificity = 98.7% [95% CI, 96.7%–99.8%], accuracy = 91.5% [95% CI, 88.2%–94.0%]) (Fig. 2; Table 3). The performance metric for LDBio test for a Bordier test threshold of 0.821 AU/mL, 0.9 AU/mL, and 1.0 AU/mL were as follows: sensitivity = 70.1% (95% CI, 60.0%–79.0%), specificity = 99.0% (95% CI, 97.0%–99.8%), accuracy = 91.7% (95% CI, 88.5%–94.3%); sensitivity = 70.8% (95% CI, 58.9%–81.0%), specificity = 93.6% (95% CI, 90.3%–96.1%), accuracy = 89.4% (95% CI, 86.9%–92.3%); and sensitivity = 71.0% (95% CI, 58.1%–81.87%), specificity = 91.7% (95% CI, 88.1%–94.4%), accuracy = 88.3% (95% CI, 84.7%–91.4%), respectively (Table S2). The LDBio test was positive for 68 (70.1%) CPA patients (*P* = 0.5000) and 3 (1.0%) non-CPA patients (*P* = 0.0104) (sensitivity = 69.4% [95% CI 59.3%–78.3%], specificity = 98.7% [95% CI 96.7%–99.8%]) (Fig. 2B; Table 3). The Bordier *Aspergillus* IgG distribution and the results of the LDBio test are shown in Fig. 2B. Of the 97 serum samples tested in the CPA patient group, 68 tested positive with the LDBio test, with a sensitivity of 69.4% (95% CI, 59.3%–78.3%). In the non-CPA group, 286 of the 289 sera tested negative with the LDBio test, with a specificity of 98.7% (95% CI, 96.7%–99.8%) (Table 3). The gold standard (Bordier) test was positive for 98 samples, of which 97 were confirmed CPA serum samples tested with 100% sensitivity (95% CI, 96.3%–100%) using a cutoff value of 0.8 AU/mL (Table 3). Comparison of the two tests showed the LDBio test to have lower sensitivity than the Bordier test for the detection of *Aspergillus* antibodies (McNemar’s *P* value <0.0001). However, the LDBio and Bordier tests showed no significant difference in specificity (McNemar’s *P* value = 0.6171) (Table 3). The results were in agreement for 353 of the 386 samples tested (91.5%) between the two tests, with a Cohen’s kappa coefficient of 0.75 (95% CI, 0.67–0.83), indicating good agreement between the LDBio and Bordier test results. Youden’s *J* statistic calculated for the LDBio test results indicated a good balance between sensitivity and specificity, and the test had a high diagnostic odds ratio of 215.3 (Table 3). Using other cutoff values of 0.821 AU/mL (optimal cutoff for Nigerians)(16), 0.9 AU/mL, and 1.0 AU/mL, the sensitivity of the Bordier test for this data set was calculated to be 100% (95% CI, 100%–100%), 74.2% (95% CI, 64.4%–82.6%), and 63.9% (95% CI, 53.5%–73.4%), respectively, with the LDBio test showing a significantly lower proportion than the Bordier test for CPA detection at cutoff values of 0.8 and 0.821 AU/mL (Table S2).

**TABLE 3:** Summary of the LDBio and Bordier test results for the sera of 386 patients^*a*^

Performance metric	Test (no. of serum samples, *n* = 386)		*P* value^*b*^
	LDBio	Bordier	
	Non-CPA = 315, CPA = 71	Non-CPA = 288, CPA = 98	
Accuracy (%)	91.5 (88.2–94.0)	99.74 (98.6–99.9)	0.0357*
Sensitivity (%)	69.4 (59.3–78.3)	100 (96.3–100)	<0.0001*
Specificity (%)	98.7 (96.7–99.8)	99.7 (98.1–99.9)	0.6171
Positive predictive value (PPV) (%)	95.8 (88–98.6)	98.9 (93.2–99.9)	0.4536
Negative predictive value (NPV) (%)	90.5 (87.6–92.8)	100 (98.7–100)	0.0189*
Positive likelihood ratio (PLR)	66.6 (21.5–206.9)	289 (40.9–2044.8)	0.3863
Negative likelihood ratio (NLR)	0.3 (0.23–0.42)	0	0.0294*
Area under the curve (AUC)	0.931	0.9949	
Youden’s index	0.681	0.997	
Diagnostic odds ratio (DOR)	215.3	∞	
Cohen’s kappa (*K*)	0.75 (0.67–0.83)	0.99 (0.98–1.00)	0.0334*
Level of agreement (%)	91.5	99.7	

^*a*^ Results are reported versus the recommended threshold of 0.8 AU/mL. Youden’s index = sensitivity + specificity − 1. Very large values = ∞. A PLR > 10 and an NLR < 0.1 are recommended to provide convincing diagnostic evidence. Hence, the LDBio test at a cutoff of 0.8 AU/mL is suboptimal because of a high NLR > 0.1. The diagnostic odds ratio (DOR) = (TP x TN)/(FP x FN) = 215.3 = the overall diagnostic accuracy. The DOR indicates the odds of a positive test for those with disease relative to the odds of a positive test for those without disease. As the DOR of the LDBio test >1, it has a very high capacity to discriminate between non-CPA (healthy) and CPA (sick) patients. We used Cohen’s kappa to measure the level of agreement between the LDBio and Bordier tests. The Cohen’s kappa was 0.68 (*P* value = 0.0334), indicating substantial agreement between the LDBio and Bordier test results.

^*b*^ * means statistically significant <0.05.

The false positive rate (FPR) for LDBio test in all types of CPA was 1.04%. The FPR for Bordier test in CCPA, CFPA, and PA was constant at 0.35%. Bordier test has lower FPR than LDBio test (Table 4). The sensitivity of the LDBio test for CCPA, CFPA, and PA was 64.4%, 75.0%, and 75.0%, respectively (Table 4).

**TABLE 4:** Sensitivity of the LDBio and Bordier tests according to the type of CPA (*N* = 386)^*a*^

Type of CPA	Error matrix				FPR	FNR	Sens	Spec	PPV	NPV	ACC
CCPA	LDBio	CPA									
			Neg	Pos	1.04%	35.6%	64.44%	98.96%	90.62%	94.70%	94.30%
		Neg	286	16			95% CI	95% CI	95% CI	95% CI	95% CI
		Pos	3	29			48.8, 78.1	97, 99.8	75.4, 96.8	92.3, 96.4	91.3, 96.5
	Bordier		Neg	Pos	0.35%	0.0%	100.00%	99.70%	97.80%	100.00%	99.70%
		Neg	288	0			95% CI	95% CI	95% CI	95% CI	95% CI
		Pos	1	45			92.1, 100	98.1, 100	86.4, 99.7	98.7, 100	98.3, 99.7
CFPA	LDBio		Neg	Pos	1.04%	25.0%	75.00%	98.96%	91.70%	96.30%	95.80%
		Neg	286	11			95% CI	95% CI	95% CI	95% CI	95% CI
		Pos	3	33			59.7, 86.8	97, 99.8	77.9, 97.2	93.9, 97.8	93.1, 97.7
	Bordier		Neg	Pos	0.35%	0.0%	100.00%	99.70%	97.80%	100.00%	99.70%
		Neg	288	0			95% CI	95% CI	95% CI	95% CI	95% CI
		Pos	1	44			91.9, 100	98.1, 100	86.4, 99.7	98.7, 100	98.3, 99.7
PA	LDBio		Neg	Pos	1.04%	25.0%	75.00%	98.96%	66.70%	99.30%	98.30%
		Neg	286	2			95% CI	95% CI	95% CI	95% CI	95% CI
		Pos	3	6			34.9, 96.8	97, 99.8	37.7, 86.9	97.7, 99.8	96.1, 99.5
	Bordier		Neg	Pos	0.35%	0.0%	100.00%	99.70%	88.90%	100.00%	99.70%
		Neg	288	0			95% CI	95% CI	95% CI	95% CI	95% CI
		Pos	1	8			63.1, 100	98.1, 100	53.1, 98.3	98.7, 100	98.1, 99.9

^*a*^ FPR, false positive rate; FNR, false negative rate; Sens, sensitivity; Spec, specificity; ACC, accuracy.

**Fig 3:**
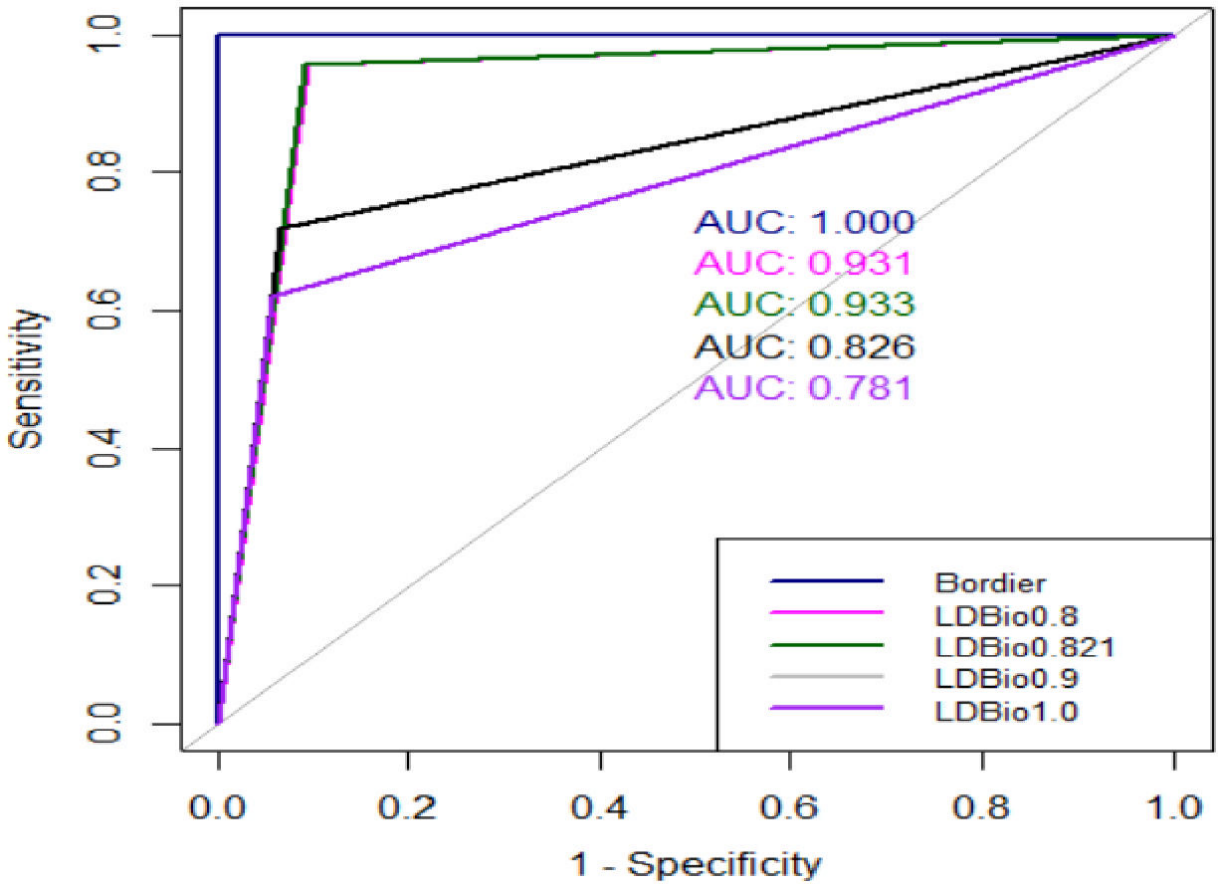
Receiver operating characteristic (ROC) curves with different positivity thresholds for the Bordier and LDBio tests. The Bordier test showed a sensitivity of 100% (95% CI, 96.3%–100%), a specificity of 99.7% (95% CI, 98.1%–99.9%), and an accuracy of 99.7% (95% CI, 99.6%–99.9%). The LDBio test showed a sensitivity of 69.4% (95% CI, 59.3%–78.3%), a specificity of 98.7% (95% CI, 96.7%–99.8%), and an accuracy of 91.5% (95% CI, 88.2%–94.0%).

We compared the accuracy of the LDBio test to that of the Bordier test using the AUC of receiver operating characteristic (ROC) curves. The LDBio test had AUCs of 0.931, 0.933, 0.826, and 0.781 for cutoffs of 0.8, 0.821, 0.9, and 1.0 AU/mL, respectively, whereas the AUCs of the Bordier test were 0.995, 1.000, 0.960, and 0.946 (Fig. 3). The profile of the performance of the LDBio test for the different thresholds of the Bordier test is presented in Fig. S1. The detection of CPA by the LDBio test decreased as the threshold increased. The optimal performance of the LDBio test occurred at a cutoff of 0.821 AU/mL with an accuracy of 91.7% (Fig. 3; Table S2).

## DISCUSSION

The diagnosis of CPA is challenging due to the heterogeneity of symptoms and its similarity to other pulmonary diseases, notably, TB. Patients with CPA are often misdiagnosed as having smear/gene Xpert-negative TB due to the similarities in symptoms and/or chest imaging, especially in TB-endemic countries. A recent review by Ekeng et al. reported 18 cases of aspergillosis misdiagnosed as TB. Of the 18, nine were cases of CPA misdiagnosed as TB; the others were allergic bronchopulmonary aspergillosis, *Aspergillus* spondylitis, and intracranial aspergilloma (19). A study from Nigeria by Oladele et al. reported a CPA prevalence of 8.7% among 208 smear-negative TB patients (14). In another study from Brazil, 15 (7%) cases of pulmonary mycoses were identified among 213 patients being managed for smear-negative TB. Of the 15 cases, 10 consisted of aspergillosis, 3 of paracoccidioidomycosis, and 1 each of histoplasmosis and cryptococcosis (20). Aside from mimicking TB, CPA also occurs as a post-TB sequela. Among 38 cases of CPA identified in a study from Vietnam, 10 were previously treated once for recurrent TB and three were treated three times (21). Another study from India reported a CPA prevalence of 57% among patients with post-TB sequelae (22). In yet another study from Britain, 25% of 544 post-TB patients were positive for IgM and IgG *Aspergillus* antibodies, 14% had aspergillomas, and 10% had precipitins without evidence of an aspergilloma after 1 year (23). As observed in this study, one of the major challenges for the clinical and radiological diagnosis of chronic pulmonary aspergillosis is the proteiform presentation of the disease with different types such as CCPA, CFPA, and PA, highlighting the need for relevant diagnostic tools.

Given the current situation, there is a dire need for an affordable and readily accessible point-of-care device like the LDBio ICT LFA test, especially in countries with a high TB burden, to enable prompt diagnosis, early initiation of therapy, and improved clinical outcomes. In addition, the availability of the LDBio test would improve CPA case detection, help in ascertaining the burden of CPA, and improve the awareness and diagnosis of CPA. It is simple, easy to use, reproducible, and inexpensive relative to other serological diagnostic techniques, such as Elisa, immunoprecipitation, and counterimmunoelectrophoresis, among others. Nigeria is currently ranked first in Africa and sixth globally among countries with a high TB burden (24) and thus has a significant population at risk for CPA, hence the need for validation of the LDBio test in our setting.

Relative to the gold standard (Bordier *Aspergillus* IgG), which has a sensitivity of 100% and specificity of 99.7%, the LDBio LFA showed a sensitivity of 69.4% with a high specificity (98.7%). However, it is important to highlight that the sensitivity of the test varies according to the CPA type and the species of *Aspergillus* responsible for the disease. Although there is a limited number of evaluations, the performance of the LDBio ICT LFA in this index study in Nigeria was slightly lower compared to other studies. Field evaluations from Indonesia and the UK reported a sensitivity of 85% and 91.6%, respectively, among the CPA population (13, 25). Besides CPA, IgG detection is also a criterion for the diagnosis of allergic broncho-pulmonary aspergillosis (ABPA) (26), and this test was also evaluated in allergic populations to *Aspergillus.* Results showed a sensitivity and a specificity from 0% and 96.4%, respectively, in Uganda with both ABPA and severe allergic (27) to 90.6% and 87.2%, respectively, in diagnosing ABPA in the United Kingdom (28). More recently, an Indian study confirmed the good performance of the test in ABPA patients, with a sensitivity of 84.9% and a specificity of 82.9% (29). Such varying performance may be attributed to (i) the absence of cross-reactivity with non-*fumigatus Aspergillus* species (13), supported by data from other studies conducted in LMIC that revealed other *Aspergillus* species to be commonly isolated in culture (14, 30), (ii) levels of *Aspergillus* IgG antibodies in serum below the limit of detection, and (iii) differences in the antigen mixtures present in the kits (13).

### Conclusion

The point-of-care test LDBio ICT LFA used in combination with other investigations, including cultures and radiology, should improve the diagnosis of CPA, especially in resource-poor settings and TB-endemic countries where missed or delayed diagnoses of CPA are common. More studies in Africa are needed to validate the performance of the LDBio ICT LFA among the CPA population to ascertain its usefulness in diagnosing CPA or ABPA.

## Supplementary Material

Reviewer comments

## SUPPLEMENTAL MATERIAL

Figure S1 spectrum.01533-24-s0001.tiff Distribution of the LDBio diagnosis for the different cutoffs of the Bordier test.

Tables S1 and S2 spectrum.01533-24-s0002.docx Contingency tables of the tests and performances according to the thresholds.

OPEN PEER REVIEW reviewer-comments.pdf An accounting of the reviewer comments and feedback.

ASM does not own the copyrights to Supplemental Material that may be linked to, or accessed through, an article. The authors have granted ASM a non-exclusive, world-wide license to publish the Supplemental Material files. Please contact the corresponding author directly for reuse.
