# Supplementary figures and images for: Evaluation of the LDBio ICT IgG/IgM lateral flow assay versus the Bordier Elisa assay for the diagnosis of chronic pulmonary aspergillosis in Nigeria
Source: Microbiol Spectr. 2025 Feb 6;13(3):e01533-24. doi: 10.1128/spectrum.01533-24 (PMC11878091; doi:10.1128/spectrum.01533-24)

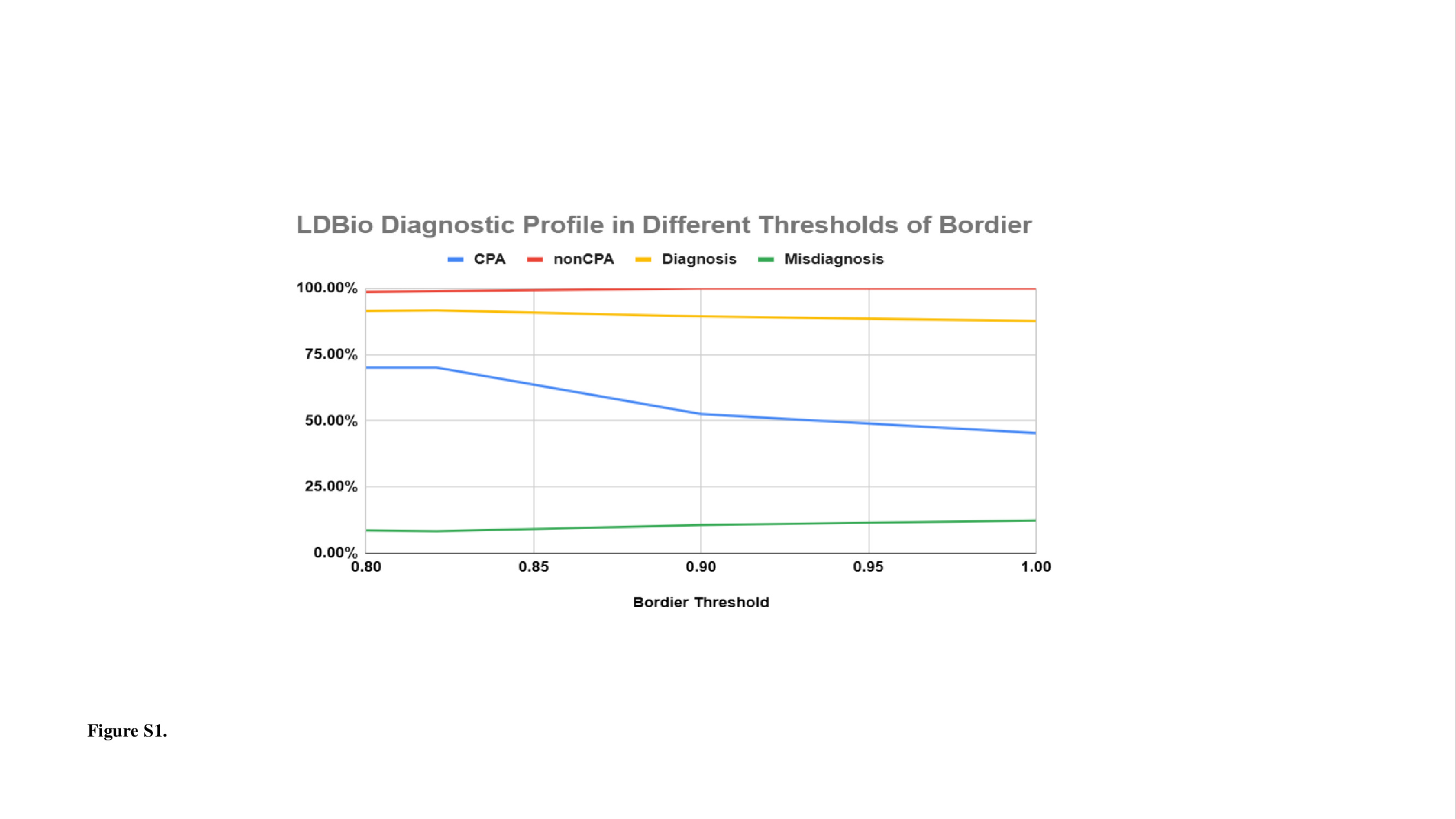

Supplement: Figure S1 — Distribution of the LDBio diagnosis for the different cutoffs of the Bordier test. [file spectrum.01533-24-s0001.tiff]
