# Supplementary material for: Evaluation of the LDBio ICT IgG/IgM lateral flow assay versus the Bordier Elisa assay for the diagnosis of chronic pulmonary aspergillosis in Nigeria
Source: Microbiol Spectr. 2025 Feb 6;13(3):e01533-24. doi: 10.1128/spectrum.01533-24 (PMC11878091; doi:10.1128/spectrum.01533-24)
Supplement: Tables S1 and S2 — Contingency tables of the tests and performances according to the thresholds. [file spectrum.01533-24-s0002.docx]

**Table S1. Contingency table of diagnostic tests**

| Cut-off = 0.8 AU/mL | Bordier = Neg | | Bordier = Pos | |
| --- | --- | --- | --- | --- |
|  | LDBio = Neg | LDBio = Pos | LDBio = Neg | LDBio = Pos |
| non CPA | 285 | 3 | 0 | 0 |
| CPA | 1 | 0 | 29 | 68 |
| Cut-off = 0.821 AU/mL | Bordier = Neg | | Bordier = Pos | |
|  | LDBio = Neg | LDBio = Pos | LDBio = Neg | LDBio = Pos |
| non CPA | 286 | 3 | 0 | 0 |
| CPA | 0 | 0 | 29 | 68 |

Bordier misclassified one non-CPA sample as CPA with a cutoff of 0.8 AU/mL. All non-CPA samples were correctly classified using a cutoff of 0.821 AU/mL.

**Table S2. Summary of the LDBio test results for the different thresholds of the Bordier test for the serum samples of 386 patients.**

| **Performance metric** | **LDBio performance for the Bordier test thresholds** | | |
| --- | --- | --- | --- |
|  | **0.821 AU/ml** | **0.9 AU/ml** | **1.0 AU/ml** |
|  | **Neg = 289, Pos = 97** | **Neg = 314, Pos = 72** | **Neg = 324, Pos = 62** |
| **Accuracy (%)** | 91.7 (88.5 - 94.3) | 89.4 (86.9 - 92.3) | 88.3 (84.7 - 91.4) |
| **Sensitivity (%)** | 70.1 (60.0 – 79.0) | 70.8 (58.9 - 81.0) | 71.0 (58.1 – 81.8) |
| **Specificity (%)** | 99.0 (97.0 – 99.8) | 93.6 (90.3 - 96.1) | 91.7 (88.1 - 94.4) |
| **PPV (%)** | 95.8 (88 – 98.6) | 71.8 (61.9 - 80.0) | 62.0 (52.3 – 70.8) |
| **NPV (%)** | 90.8 (87.9 – 93.1) | 93.3 (90.7 - 95.3) | 94.3 (91.8 - 96.1) |
| **PLR** | 67.5 (21.8 – 209.7) | 11.1 (7.1 - 17.4) | 8.5 (5.7 - 12.6) |
| **NLR** | 0.3 (0.2 - 0.4) | 0.3 (0.2 - 0.5) | 0.3 (0.2 - 0.5) |
| **AUC** | 0.933 | 0.826 | 0.781 |
| **Youden's Index** | 0.691 | 0.644 | 0.627 |
| **DOR** | 223.5 | 35.7 | 26.9 |
| **Cohen's Kappa (K)** | 0.7582 | 0.6481 | 0.5916 |

**Figure S1. Distribution of the LDBio diagnosis for the different cutoffs of the Bordier test.** The detection of CPA by the LDBio test decreased as the threshold of the of Bordier test increased. The specificity was above 95% for all thresholds. The misdiagnosis of CPA by the LDBio test increased slightly as the Bordier test cutoffs increased. There was a relative decrease in the proportion of diagnosis (Accuracy) for the LDBio test as the threshold increased.
