# Supplementary material for: Evaluation of the LDBio ICT IgG/IgM lateral flow assay versus the Bordier Elisa assay for the diagnosis of chronic pulmonary aspergillosis in Nigeria
Source: Microbiol Spectr. 2025 Feb 6;13(3):e01533-24. doi: 10.1128/spectrum.01533-24 (PMC11878091; doi:10.1128/spectrum.01533-24)
Supplement: Reviewer comments [file reviewer-comments.pdf]

AMERICAN SOCIETY FOR MICROBIOLOGY | Microbiology Spectrum

Evaluation of the LDBio ICT IgG/IgM lateral flow assay versus the Bordier Elisa assay for the diagnosis of chronic pulmonary aspergillosis in Nigeria

Oluwaseyi Balogun, Rita Oladele, Olawale Ajibola, Adeyinka Davies, Theophilus Fashanu, Augustina Nwosu, Bassey Ekeng, and Jean-Pierre Gangneux

Corresponding Author(s): Jean-Pierre Gangneux, Universite de Rennes

Review Timeline:

Submission Date:	June 30, 2024
Editorial Decision:	September 19, 2024
Revision Received:	November 11, 2024
Accepted:	December 30, 2024

Editor: Christina Cuomo

Reviewer(s): Disclosure of reviewer identity is with reference to reviewer comments included in decision letter(s). The following individuals involved in review of your submission have agreed to reveal their identity: Oxana Munteanu (Reviewer #2)

Transaction Report:

(Note: With the exception of the correction of typographical or spelling errors that could be a source of ambiguity, letters and reports are not edited. The original formatting of letters and referee reports may not be reflected in this compilation.)

DOI: https://doi.org/10.1128/spectrum.01533-24

Re: Spectrum01533-24 (Evaluation of the LDBio ICT IgG/IgM lateral flow assay versus the Bordier Elisa assay for the diagnosis of chronic pulmonary aspergillosis in Nigeria)

Dear Prof. Jean-Pierre Gangneux:

Thank you for the privilege of reviewing your work. Below you will find my comments, instructions from the Spectrum editorial office, and the reviewer comments.

First, my apologies with the delay with your manuscript. One of the reviewers asked for extra time and then failed to return a review so a new reviewer was recruited. Two reviewers have now provided feedback that I would like you to address in a revision.

Please return the manuscript within 60 days; if you cannot complete the modification within this time period, please contact me. If you do not wish to modify the manuscript and prefer to submit it to another journal, notify me immediately so that the manuscript may be formally withdrawn from consideration by Spectrum.

Revision Guidelines
To submit your modified manuscript, log into the submission site at https://spectrum.msubmit.net/cgi-bin/main.plex. Go to Author Tasks and click the appropriate manuscript title to begin. The information you entered when you first submitted the paper will be displayed; update this as necessary. Note the following requirements:

• Upload point-by-point responses to the issues raised by the reviewers in a file named "Response to Reviewers," NOT in your cover letter.
• Upload a compare copy of the manuscript (without figures) as a "Marked-Up Manuscript" file.
• Upload a clean .DOC/.DOCX version of the revised manuscript and remove the previous version.
• Each figure must be uploaded as a separate, editable, high-resolution file (TIFF or EPS preferred), and any multipanel figures must be assembled into one file.
• Any supplemental material intended for posting by ASM should be uploaded with their legends separate from the main manuscript. You can combine all supplemental material into one file (preferred) or split it into a maximum of 10 files with all associated legends included.

For complete guidelines on revision requirements, see our Submission and Review Process webpage. Submission of a paper that does not conform to guidelines may delay acceptance of your manuscript.

Data availability: ASM policy requires that data be available to the public upon online posting of the article, so please verify all links to sequence records, if present, and make sure that each number retrieves the full record of the data. If a new accession number is not linked or a link is broken, provide Spectrum production staff with the correct URL for the record. If the accession numbers for new data are not publicly accessible before the expected online posting of the article, publication may be delayed; please contact production staff (Spectrum@asmusa.org) immediately with the expected release date.

Publication Fees: For information on publication fees and which article types are subject to charges, visit our website. If your manuscript is accepted for publication and any fees apply, you will be contacted separately about payment during the production process; please follow the instructions in that e-mail. Arrangements for payment must be made before your article is published.

ASM Membership: Corresponding authors may join or renew ASM membership to obtain discounts on publication fees. Need to upgrade your membership level? Please contact Customer Service at Service@asmusa.org.

The ASM Journals program strives for constant improvement in our submission and publication process. Please tell us how we can improve your experience by taking this quick Author Survey.

Thank you for submitting your paper to Spectrum.

Sincerely,
Christina Cuomo
Editor
Microbiology Spectrum

Reviewer #1 (Comments for the Author):

This manuscript evaluates the application of the LDBio lateral flow assay for Aspergillus fumigatus compared to the high sensitivity Bordier Elisa assay for clinical samples in Nigeria. The authors use well established approaches to compare a clinical

cohort with both methods, and benchmark the detection by both methods. Overall there is higher sensitivity with the Bordier method however the authors provide data supporting the use of the LDBio test, which with advantages of cost and accessibility could support wider diagnosis of aspergillosis in settings where the Elisa test is not available.

The manuscript would be improved first by describing some background on these clinical cases; how they were confirmed to be CPA (what data used for the ERS/ESCMIID review) and ideally a summary of any pulmonary health conditions that would provide background on the cohort tested.

Secondly, the presentation of results was difficult to follow where results were summarized across several figures/tables (e.g. lines 126-131). If AUC variation was a primary way to evaluate the tests, it seemed sporadically presented, appearing for only one test in an individual figure (1d- Bordier, 2- LDBio with AUC) or table (table 3- LDbio). Also Figure 1C does not appear to be clearly described.

In the conclusions, it would be helpful to be clear if the other studies of the LDBio kit used different thresholds, and it may help for the authors to comment more on whether that should continue to be a variable continuing to test that for regions in Africa, or if species typing should be incorporated to help confirm if that is the reason for false positives as is suggested.

Minor comments
Figure 1A - 97 CPA Pos are shown for Bordier, however the text lists 98 (lines 126 and 137) - can the authors clarify this difference.
Figure 2 legend "The Border test" -> "The Bordier test"
Line 239: Are the numbers for Uganda correct (sensitivity of 0%)? If so, what explains that failure to detect - all a different species in Uganda?

Reviewer #2 (Comments for the Author):

Dear authors,
I have read the submitted article with great interest. Overall, the article is well constructed and provides valuable insights into the comparative performance of the LDBio ICT IgG/IgM lateral flow assay and the Bordier ELISA assay for the diagnosis of chronic pulmonary aspergillosis (CPA). However, there are some areas where the discussion could be further developed to improve the understanding of the study results and their implications.
Reliable diagnostics contribute to a better understanding of the prevalence of CPA in different populations and improve surveillance efforts, which is important for public health planning and resource allocation. The evaluation of newer diagnostic technologies such as the LDBio ICT IgG/IgM assay against established methods such as ELISA promotes innovation and advancement in diagnostic methods. Such comparative studies contribute to the standardization of CPA diagnostic protocols, ensuring consistent and high-quality testing in different settings. Point-of-care assays that are reliable and easy to use can have a significant impact on the management of CPA in resource-limited settings where access to sophisticated laboratory infrastructure may be limited. Understanding the false positive and false negative rates of each assay is critical to improving diagnostic accuracy and reducing misdiagnosis.
In order to improve the evaluation of the LDBio ICT IgG/IgM Lateral Flow assay compared to the Bordier ELISA assay for the diagnosis of chronic pulmonary aspergillosis (CPA), the following detailed points and comments are proposed to be included:
Detailed Description of the CPA Patient Population
Types of CPA and Underlying Lung Pathology
• Patient Stratification: Describe the different types of CPA present in the study cohort, such as chronic cavitary pulmonary aspergillosis (CCPA), chronic fibrosing pulmonary aspergillosis (CFPA), Aspergillus nodules, and aspergilloma.
• Underlying Conditions: Provide detailed information on the underlying pulmonary pathologies in the patient group, including post-tuberculosis sequelae, bronchiectasis, sarcoidosis, COPD, and others.
Understanding False Negative Cases and Type-Specific Sensitivity
• Type-Specific Sensitivity: Investigate whether certain types of CPA are more susceptible to false negatives with the LDBio assay.
Comment on False Positive Rates in the Bordier ELISA Assay
• Comparison with Existing Literature: The finding that only 17 out of 289 healthy subjects (6%) had false positive results using the Bordier ELISA assay requires comment in the discussion. This is significantly lower than the 30% prevalence reported in the study by Salzer H et al., "Aspergillus-specific IgG antibodies for diagnosing chronic pulmonary aspergillosis compared to the reference standard." Consider factors that might contribute to this discrepancy, such as differences in study populations, diagnostic criteria, or assay performance.
- Conclusions should be revised
- The recommendation for additional studies, particularly in Africa, to determine the performance of the test in CPA populations is valid, but should be framed as a discussion point rather than a conclusion supported by the results of the current study.

Evaluation of the LDBio ICT IgG/IgM lateral flow assay versus the Bordier Elisa assay for the diagnosis of chronic pulmonary aspergillosis in Nigeria

We warmly thank the reviewer and Editor for their constructive comments and important remarks and suggestions on this manuscript.
Please see below our point-by-point responses to the issues raised by the reviewers

Reviewer #1 (Comments for the Author):

This manuscript evaluates the application of the LDBio lateral flow assay for Aspergillus fumigatus compared to the high sensitivity Bordier Elisa assay for clinical samples in Nigeria. The authors use well established approaches to compare a clinical cohort with both methods, and benchmark the detection by both methods. Overall there is higher sensitivity with the Bordier method however the authors provide data supporting the use of the LDBio test, which with advantages of cost and accessibility could support wider diagnosis of aspergillosis in settings where the Elisa test is not available.
We thank the reviewer for his kind general comment.

The manuscript would be improved first by describing some background on these clinical cases; how they were confirmed to be CPA (what data used for the ERS/ESCMIID review) and ideally a summary of any pulmonary health conditions that would provide background on the cohort tested.
We agree with this comment (similar to reviewer's #2 comment) and take the opportunity to give much more details on the socio-demographic, clinical characteristics of the patients and radiological findings:
- Definition of the disease is mentioned p.3
- We included two new tables (Tables 1 & 2)
- We included a new figure (Figure 1) that reports Chest X-ray findings according to the CPA type.

Secondly, the presentation of results was difficult to follow where results were summarized across several figures/tables (e.g. lines 126-131). If AUC variation was a primary way to evaluate the tests, it seemed sporadically presented, appearing for only one test in an individual figure (1d- Bordier, 2- LDBio with AUC) or table (table 3- LDbio). Also Figure 1C does not appear to be clearly described.
The Result section has been modified according reviewers's 1&2 comments (see below response to reviewer 2).

In the conclusions, it would be helpful to be clear if the other studies of the LDBio kit used different thresholds, and it may help for the authors to comment more on whether that should continue to be a variable continuing to test that for regions in Africa, or if species typing should be incorporated to help confirm if that is the reason for false positives as is suggested.

Regarding the comment on the threshold, we respectfully remind the reviewer that the LDBio LFA kit is a qualitative rapid test that is either positive or negative. We can only discuss on the limit of detection but not on a threshold of interpretation.
Regarding the comment of Aspergillus species that can impact the sensitivity of the test, we agree with the reviewer that's an important point and discuss it at the end of the Discussion section

Minor comments
Figure 1A - 97 CPA Pos are shown for Bordier, however the text lists 98 (lines 126 and 137) - can the authors clarify this difference.
Thank you for this remark. We have now clarified the text : "98 patients had positive Aspergillus IgG levels (≥ 0.8 AU/ml). Among them 97 had radiological features consistent with CPA…"

Figure 2 legend "The Border test" -> "The Bordier test"
Corrected

Line 239: Are the numbers for Uganda correct (sensitivity of 0%)? If so, what explains that failure to detect - all a different species in Uganda?
Yes they are correct. However the population tested in this work was not CPA but allergic patients with most of them presenting with severe asthma with fungal sensitization and a low number of ABPA. The text has now been clarified.

Reviewer #2 (Comments for the Author):

Dear authors,
I have read the submitted article with great interest. Overall, the article is well constructed and provides valuable insights into the comparative performance of the LDBio ICT IgG/IgM lateral flow assay and the Bordier ELISA assay for the diagnosis of chronic pulmonary aspergillosis (CPA). However, there are some areas where the discussion could be further developed to improve the understanding of the study results and their implications. Reliable diagnostics contribute to a better understanding of the prevalence of CPA in different populations and improve surveillance efforts, which is important for public health planning and resource allocation. The evaluation of newer diagnostic technologies such as the LDBio ICT IgG/IgM assay against established methods such as ELISA promotes innovation and advancement in diagnostic methods. Such comparative studies contribute to the standardization of CPA diagnostic protocols, ensuring consistent and high-quality testing in different settings. Point-of-care assays that are reliable and easy to use can have a significant impact on the management of CPA in resource-limited settings where access to sophisticated laboratory infrastructure may be limited. Understanding the false positive and false negative rates of each assay is critical to improving diagnostic accuracy and reducing misdiagnosis.
We warmly thank the reviewer for his kind general comment.

In order to improve the evaluation of the LDBio ICT IgG/IgM Lateral Flow assay compared to the Bordier ELISA assay for the diagnosis of chronic pulmonary aspergillosis (CPA), the following detailed points and comments are proposed to be included:

Detailed Description of the CPA Patient Population

Types of CPA and Underlying Lung Pathology

• Patient Stratification: Describe the different types of CPA present in the study cohort, such as chronic cavitary pulmonary aspergillosis (CCPA), chronic fibrosing pulmonary aspergillosis (CFPA), Aspergillus nodules, and aspergilloma.

• Underlying Conditions: Provide detailed information on the underlying pulmonary pathologies in the patient group, including post-tuberculosis sequelae, bronchiectasis, sarcoidosis, COPD, and others.

We agree with this comment (similar to reviewer's #1 comment) and take the opportunity to give much more details on the socio-demographic, clinical characteristics of the patients and radiological findings:

- Definition of the disease is mentioned p.3
- We included two new tables (Tables 1 & 2)
- We included a new figure (Figure 1) that reports Chest X-ray findings and IgG titles according to the CPA type.

Understanding False Negative Cases and Type-Specific Sensitivity

• Type-Specific Sensitivity: Investigate whether certain types of CPA are more susceptible to false negatives with the LDBio assay.

Thank you for this valuable comment. We added new statistical results on the sensitivity and specificity according to the CPA type in Table 4. This point is discussed now in the Discussion section since the analysis interestingly showed a lower performance of the LDBio test in CCPA compared to CFPA and PA.

Comment on False Positive Rates in the Bordier ELISA Assay

• Comparison with Existing Literature: The finding that only 17 out of 289 healthy subjects (6%) had false positive results using the Bordier ELISA assay requires comment in the discussion. This is significantly lower than the 30% prevalence reported in the study by Salzer H et al., "Aspergillus-specific IgG antibodies for diagnosing chronic pulmonary aspergillosis compared to the reference standard." Consider factors that might contribute to this discrepancy, such as differences in study populations, diagnostic criteria, or assay performance.

We indeed know this study with an abnormally high false-positive rate that is far from the real life in our experience. We did not mention it because this can disturb the debate on the interest of IgG detection for the diagnosis of CPA since this analysis was performed using the IgG ImmunoCap assay with its controversial threshold of positivity of 50 mgA/L

Conclusions should be revised

• The recommendation for additional studies, particularly in Africa, to determine the performance of the test in CPA populations is valid, but should be framed as a discussion point rather than a conclusion supported by the results of the current study.

We agree with the reviewer that we need evaluation from different countries because the Aspergillus species responsible may vary, and it is now more extensively discussed in this revised version. But we respectfully propose to also keep a sentence in the conclusion as it is a key point from our point of view.

Re: Spectrum01533-24R1 (Evaluation of the LDBio ICT IgG/IgM lateral flow assay versus the Bordier Elisa assay for the diagnosis of chronic pulmonary aspergillosis in Nigeria)

Dear Prof. Jean-Pierre Gangneux:

Your manuscript has been accepted, and I am forwarding it to the ASM production staff for publication. Your paper will first be checked to make sure all elements meet the technical requirements. ASM staff will contact you if anything needs to be revised before copyediting and production can begin. Otherwise, you will be notified when your proofs are ready to be viewed.

Data Availability: ASM policy requires that data be available to the public upon online posting of the article, so please verify all links to sequence records, if present, and make sure that each number retrieves the full record of the data. If a new accession number is not linked or a link is broken, provide production staff with the correct URL for the record. If the accession numbers for new data are not publicly accessible before the expected online posting of the article, publication may be delayed; please contact ASM production staff immediately with the expected release date.

Publication Fees: For information on publication fees and which article types have charges, please visit our website. We have partnered with Copyright Clearance Center (CCC) to collect author charges. If fees apply to your paper, you will receive a message from no-reply@copyright.com with further instructions. For questions related to paying charges through RightsLink, please contact CCC at ASM_Support@copyright.com or toll free at +1-877-622-5543. CCC makes every attempt to respond to all emails within 24 hours.

ASM Membership: Corresponding authors may join or renew ASM membership to obtain discounts on publication fees. Need to upgrade your membership level? Please contact Customer Service at Service@asmusa.org.

PubMed Central: ASM deposits all Spectrum articles in PubMed Central and international PubMed Central-like repositories immediately after publication. Thus, your article is automatically in compliance with the NIH access mandate. If your work was supported by a funding agency that has public access requirements like those of the NIH (e.g., the Wellcome Trust), you may post your article in a similar public access site, but we ask that you specify that the release date be no earlier than the date of publication on the Spectrum website.

Embargo Policy: A press release may be issued as soon as the manuscript is posted on the Spectrum Latest Articles webpage. The corresponding author will receive an email with the subject line "ASM Journals Author Services Notification" when the article is available online.

The ASM Journals program strives for constant improvement in our submission and publication process. Please tell us how we can improve your experience by taking this quick Author Survey.

Thank you for submitting your paper to Spectrum.

Sincerely,
Christina Cuomo
Editor
Microbiology Spectrum

Reviewer #1 (Comments for the Author):

The authors have thoroughly addressed my comments in this revision.

Reviewer #2 (Comments for the Author):

I am pleased to inform you that I have no further comments. I accept the article for publication as is.
